# Molecular Modeling of Triethyl Phosphate: Extension and Validation of the TraPPE Force Field

**DOI:** 10.1021/acs.jpcb.6c03259

**Published:** 2026-08-11

**Authors:** Geordy Jomon, Santiago A. Flores Roman, Andrei L. Kolesnikov, Gennady Y. Gor

**Affiliations:** † Otto H. York Department of Chemical and Materials Engineering, 122389 New Jersey Institute of Technology, Newark, New Jersey 07102, United States; ‡ Institut für Nichtklassische Chemie e.V., Leipzig 04318, Germany

## Abstract

The study of physical properties of organophosphorus chemical warfare agents (CWAs) is essential for detection, protection, decontamination, and destruction. Due to the extreme toxicity of CWAs, other organophosphorus molecules with similar chemical structures are employed in experimental studies as simulants, such as dimethyl methylphosphonate (DMMP) and diisopropyl methylphosphonate (DIMP). Triethyl phosphate (TEP) can also be considered an alternative simulant, especially because it is more accessible due to its various industrial applications. Classical molecular simulations complement the experimental research on organophosphates. Here, we present the extension of the transferable potentials for phase equilibria (TraPPE) force field to TEP. Using this model, TEP can be parametrized based on interaction parameters from similar organophosphates in conjunction with *ab initio* calculations. The model was validated by performing molecular dynamics (MD) simulations to predict liquid densities and Gibbs ensemble Monte Carlo simulations to model vapor–liquid equilibrium and to predict vapor pressures, which were compared with literature data. MD simulations were performed to predict the enthalpy of vaporization, surface tension, viscosity, and diffusivity. Additionally, we parametrized and tested trimethyl phosphate (TMP) due to its structural similarity to TEP. Our study helps assess TEP and TMP as alternative CWA simulants and provides predictions of their physical properties over a wide range of temperatures, complementing the scarce experimental data. The presented force field can be further utilized for predicting other properties based on molecular simulations, such as adsorption of TEP or TMP.

## Graphical abstract

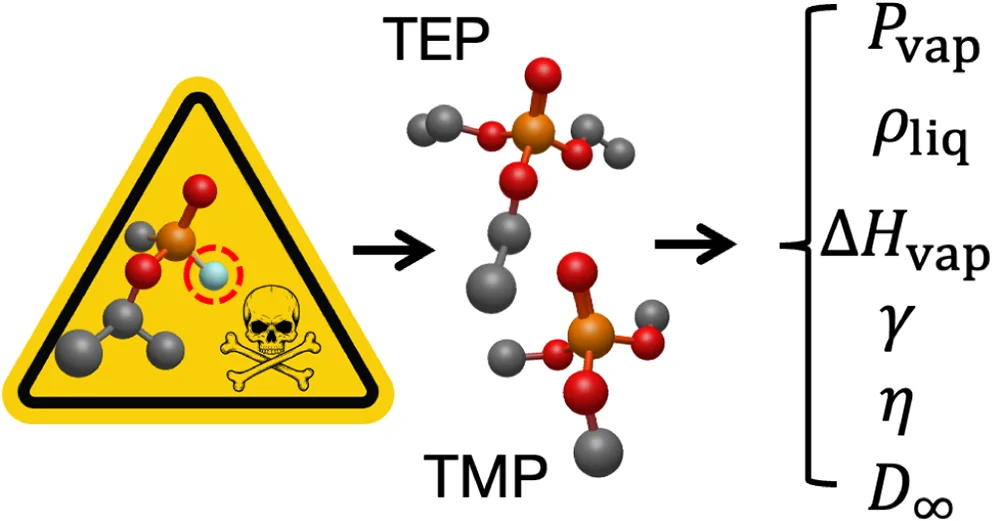

## Introduction

Organophosphorus chemical warfare agents (CWAs) are highly toxic, rapid-onset nerve agents. When absorbed through the skin or lungs, they inhibit acetylcholinesterase, leading to the hyperstimulation of muscles, glands, and the central nervous system.^1^ Even though the production, use, and stockpiling of CWAs have been banned by the 1993 Chemical Weapons Convention, they are still used by bad actors.^2^ This stimulates continuing research on CWAs focused on detection, protection, decontamination, and destruction of these agents. However, experiments on CWAs have to be very limited due to their extreme toxicity, and most experiments are carried out using nontoxic organophosphates as CWA simulants.

To have comparable physical and chemical properties, simulants should have relatively similar molecular size and structure. Figure 1 shows molecular structures of organophosphorus compounds considered in this work. The most common simulant is dimethyl methylphosphonate (DMMP).^3–7^ In recent years, there has been growing interest in alternative simulants that better represent certain properties of CWAs. In particular, diisopropyl methylphosphonate (DIMP) has been demonstrated to be advantageous when studying agent chemical decomposition.^8–14^

**Figure 1:**
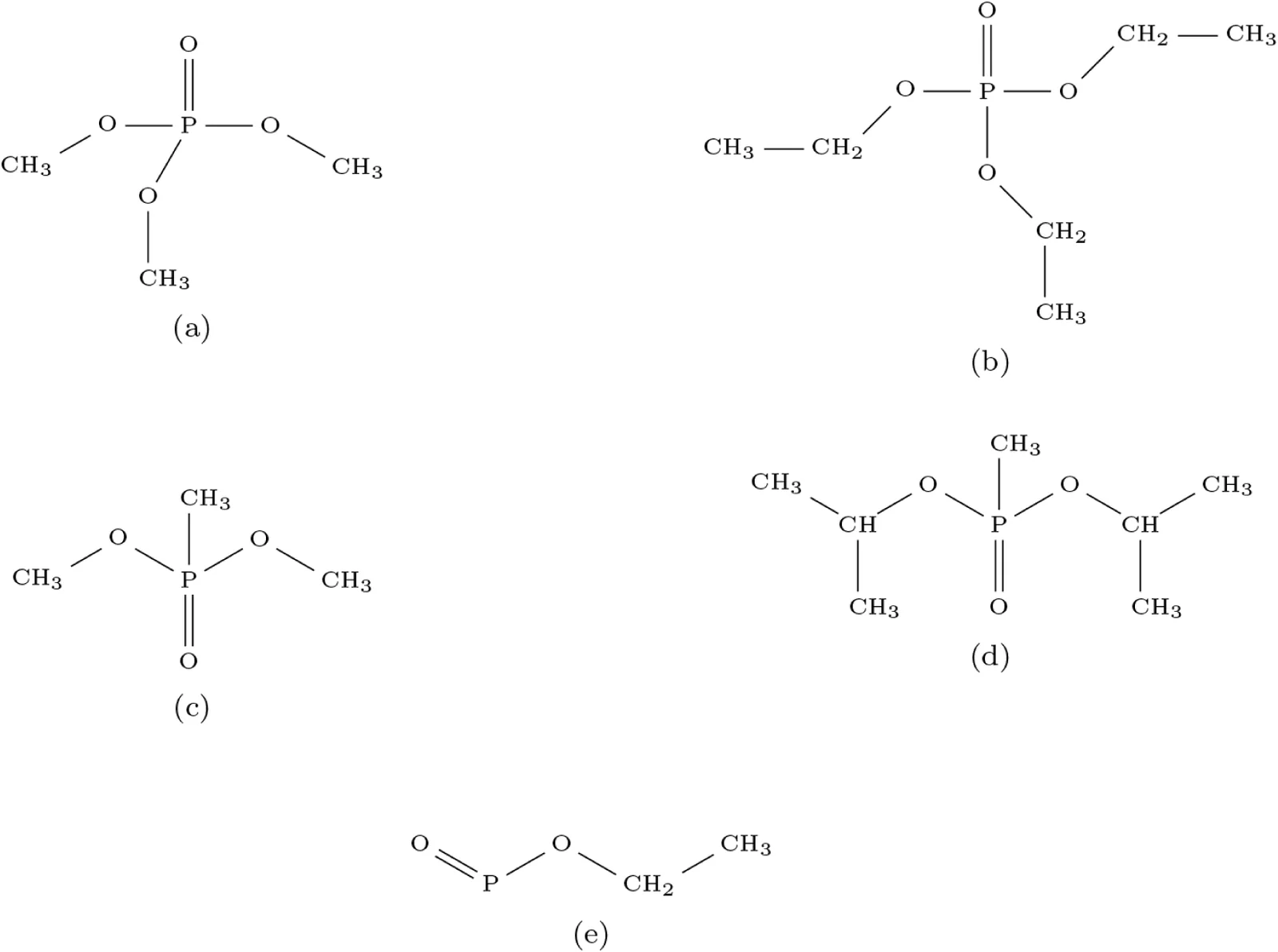
Molecular structures of organophosphorus compounds studied in this work: trimethyl phosphate—TMP (C_3_H_9_O_4_P) (a), triethyl phosphate—TEP (C_6_H_15_O_4_P) (b), dimethyl methylphosphonate—DMMP (C_3_H_9_O_3_P) (c), and diisopropyl methylphosphonate—DIMP (C_7_H_17_O_3_P) (d). Ethyl phosphinate (C_2_H_5_O_2_P) (e) is used for the potential energy scan.

Other aspects are related to properties of agents in aerosolized form. Aerosol formation is the primary mode of agent release, where parameters such as vapor pressure, the Reynolds number (which depends on viscosity), and the Weber number (which depends on surface tension) describe its formation and evolution.^15^ These are necessary for accurate atmospheric dispersion modeling of the agents.^16^ Unfortunately, DMMP and DIMP do not represent properties relevant to aerosols well. In particular, the surface tension of DMMP is noticeably higher than that of the agent, while the surface tension of DIMP is lower.^15^ Furthermore, the viscosity of liquid DIMP is significantly higher than that of the agent.^17,18^ Hence, there is a growing need to explore alternative simulants. Triethyl phosphate (TEP) emerges as a promising alternative CWA simulant, which is more readily available and cost-effective than DIMP due to its industrial use. However, relevant properties of TEP, such as liquid density,^19,20^ vapor pressure,^21^ surface tension,^22,23^ and viscosity,^19,24,25^ are scarce, often reported at a single temperature, and sometimes inconsistent.

Within the last two decades, classical molecular simulation has become an accurate tool for predicting the physical and chemical properties of fluids. Molecular simulation predictions can complement experimental measurements, especially when experiments are too challenging. For example, simulations can predict the properties of CWAs over a range of temperatures and pressures, providing a safe and reliable alternative to additional experiments with agents and to simple extrapolation of scarce experimental data. While experiments with CWA simulants are much safer and more manageable than with agents themselves, molecular simulations of simulants still offer advantages.^7,26,27^ Simulation-based predictions are more cost-efficient and free from impurities, which can significantly affect experimental measurements, e.g., viscosity.^18^

Several molecular simulation studies have focused on predicting properties of TEP.^25,28–30^ A broad molecular dynamics simulation study of organic liquids using OPLS (Optimized Potentials for Liquid Simulations) and GAFF (General Amber Force Field) included calculations for TEP,^28^ reporting liquid density, surface tension, and enthalpy of vaporization. The liquid densities calculated using OPLS were close to the reference data, while GAFF overpredicted the density significantly. The predictions for surface tension were close to that in Yaws handbook,^31^ and the enthalpy of vaporization calculated using both force fields appeared to overpredict the reference data. Das et al. used molecular dynamics simulation with the OPLS force field and studied the hydration properties of TEP.^29^ Warmińska and Ṡmiechowski, also using OPLS, focused on calculations of radial distribution functions and hydrogen bond characteristics for TEP solutions.^25^ Recently, Smith and Sega attempted to use OPLS and GAFF to predict the viscosity of liquid TEP.^30^ Their calculations with GAFF failed, while OPLS predicted values an order of magnitude higher than the experimental data. Therefore, there remains a need for reliable force fields that can accurately predict TEP properties, particularly its viscosity.

The transferable potentials for phase equilibria (TraPPE) force field^4^ has previously been used to model CWA simulants DMMP and DIMP, as well as agents of interest, successfully predicting thermodynamic^15,32^ and transport properties.^17^ The goals of this work are (i) to extend the TraPPE force field to TEP, (ii) to assess the predictive capability of the resulting force field using available experimental data, and (iii) to extend predictions of TEP properties over a broad temperature range. The predicted properties will help assess the potential of using TEP as a CWA simulant. The extension of the force field will also enable molecular simulations of another organophosphorus compound, trimethyl phosphate (TMP), allowing for an assessment of suitability as a simulant.

## Methods

### Force Field

To parametrize trimethyl phosphate (TMP) and triethyl phosphate (TEP) molecules, we have used transferable potentials for phase equilibria (TraPPE), which has been utilized previously to predict the thermodynamic and transport properties of organophosphates such as dimethyl methylphosphonate (DMMP), diisopropyl methylphosphonate (DIMP), and chemical warfare agents (CWAs).^4,32^ In the TraPPE model, energy interactions are defined as a sum of nonbonded and bonded interactions. The 12–6 Lennard-Jones potential along with electrostatics constitutes the nonbonded interactions,

1 $$U_{\mathrm{non-bonded}}=4\epsilon_{ij}\left[{\left(\frac{\sigma_{ij}}{r_{ij}}\right)}^{12}-{\left(\frac{\sigma_{ij}}{r_{ij}}\right)}^{6}\right]+\frac{q_{i}q_{j}}{4\pi \varepsilon_{0}r_{ij}}$$

where σ and ϵ are Lennard-Jones (LJ) parameters, *r*_*ij*_ is the distance between interacting sites *i* and *j*, *q*_*i*_, and *q*_*j*_ are respective partial charges, and ε_0_ is the vacuum permittivity. Long-range electrostatics are calculated using the Ewald summation.^32^ Other methods such as the particle-mesh Ewald (PME) or the particle–particle particle-mesh (PPPM) were not considered, as they were not supported in the MC simulation software that we used in this work. The nonbonded interactions were included for sites separated by at least 4 bonds (1–5 terms and above). To reduce computational cost, the united atom representation for TraPPE (TraPPE-UA) is used, where the hydrogen atom in a CH_*x*_ group is not simulated explicitly, but the entire group is considered as a single interaction site. Figure 2 shows the molecules considered in this work in the UA representation. The bonded interactions in the TraPPE model include bond angle bending and dihedral rotation. TraPPE uses the fixed bond length, which saves computational time for MC simulations. The bond angle bending is defined as a harmonic potential. We also used a harmonic potential for bond stretching when performing MD simulations.^32^

**Figure 2:**
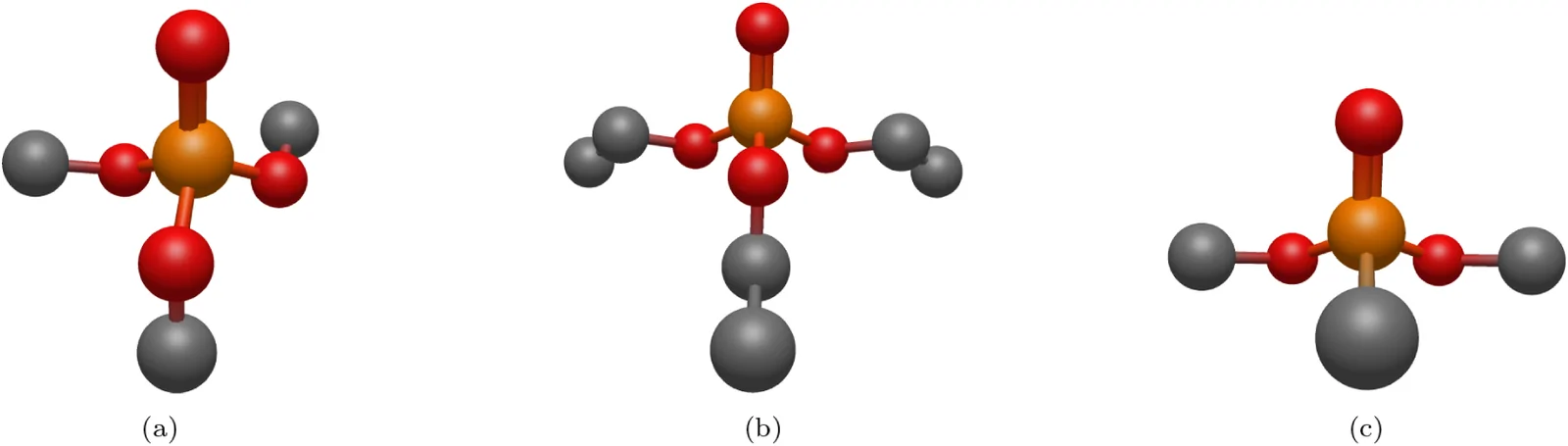
United atom representation of trimethyl phosphate—TMP (C_3_H_9_O_4_P) (a), triethyl phosphate—TEP (C_6_H_15_O_4_P) (b), and dimethyl methylphosphonate—DMMP (C_3_H_9_O_3_P) (c). Here, CH_2_ and CH_3_ groups are represented by a single interaction site. The sites P, O, CH_2_, and CH_3_ are colored orange, red, and gray, respectively.


2 $$U_{\mathrm{bonded}}=U_{\mathrm{bond}}+U_{\mathrm{angle}}+U_{\mathrm{dihedral}}$$

3 $$U_{\mathrm{bond}}=K_{\mathrm{bond}}{\left(r-r_{0}\right)}^{2}$$

4 $$U_{\mathrm{angle}}=K_{\mathrm{angle}}{\left(\theta -\theta_{0}\right)}^{2}$$

5 $$U_{\mathrm{dihedral}}=\overset{4}{\underset{i=1}{\sum }}K_{i}\left[1+\cos\left(n_{i}\tau -d_{i}\right)\right]$$

Here, *K*_bond_ and *K*_angle_ are harmonic force constants, and *r*_0_ and θ_0_ are the equilibrium bond length and angle size, respectively. The dihedral interactions are defined as a cosine series, where *K*_*i*_ is the dihedral force constant, and *d*_*i*_ is the phase shift.^17^ Using TraPPE, one can transfer the interaction parameters for different atoms and functional groups from one molecule to another.

For TMP (Figure 2b), the LJ parameters and all the nonbonding interaction parameters are transferred from the existing TraPPE model of DMMP.^32^ While the LJ parameters are transferable, the partial charges are calculated separately for each of the molecules, following the approach from refs 4 and 32. The partial charges for the electrostatic interactions were obtained from CHELPG analysis using *ab initio* calculations.^33^ The molecule was first optimized using the B3LYP/6–311++G­(d,p) level of theory. The partial charges for hydrogen atoms were summed up with the carbon atom for each functional group for the TraPPE-UA representation, and the rest were used as is. All *ab initio* calculations were performed using Gaussian 16.^34^ Table 1 shows the nonbonded interaction parameters for TMP.

**Table 1:** Nonbonded Interaction Parameters for TMP^a^

Atom	σ, Å	ϵ, kcal/mol	charge, *e*
P	4.00	0.171	1.275
O=	3.05	0.157	–0.675
O–	2.80	0.109	–0.441
CH_3_	3.75	0.195	0.242

a The σ and ϵ parameters are based on the description of DMMP,^32^ and the charges are calculated using *ab initio* CHELPG analysis.

The TEP molecule (Figure 2a) has a structure similar to that of TMP, except for additional CH_2_ groups. To account for this, we transferred the LJ interaction from TraPPE definition of ethyl methyl ether, which has an O–CH_2_–CH_3_ chain similar to TEP.^35^ The nonbonded parameters involving CH_2_ were obtained from a CWA.^32^ However, there is still one dihedral angle that is not defined for TraPPE, namely P–O–CH_2_–CH_3_. To parametrize it, we follow the procedure utilized by Emelianova and Gor for the TraPPE force field for isocyanates.^33^ We do not use TEP for this procedure since it is a large molecule, which causes steric hindrance with the other two P–O–CH_2_–CH_3_ chains, resulting in discontinuities in the scan. Instead, we perform the scan on ethyl phosphinate (Figure 1e), a small linear molecule containing the P–O–CH_2_–CH_3_ dihedral. We perform a 72-point relaxed potential energy scan where the dihedral angle is rotated by 5°, followed by an optimization for each point. To account for this optimization, we isolated the dihedral contribution from the potential energy scan. This was done by subtracting the 1–5 LJ, 1–5 electrostatics, bond, angle, and the remaining O=P–O–CH_2_ dihedral energy contributions at each step of the scan.

The resulting dihedral scan is fitted to the cosine dihedral form (eq 5), and transferred to TEP. For the rest of the parameters, we follow the same procedure as TMP. Table 2 shows the nonbonded parameters for TEP. Tables 3, 4, and 5 show the bonded stretch, bend, and dihedral parameters, respectively, used in both molecules.

**Table 2:** Nonbonded Interaction Parameters for TEP^a^

Atom	σ, Å	ϵ, kcal/mol	charge, *e*
P	4.00	0.171	1.225
O=	3.05	0.157	–0.668
O–	2.80	0.109	–0.510
CH_2_	3.95	0.0914	0.370
CH_3_	3.75	0.195	–0.045

a The σ and ϵ parameters are based on the description of DMMP^32^ and ethyl methyl ether (only for CH_2_).^35^ The charges are calculated using *ab initio* CHELPG analysis.

**Table 3:** Bond Stretching Parameters for TMP and TEP^a^

Bond	*K*_bond_, kcal/mol· Å	*r*_0_, Å
P=O	1003.82	1.458
P–O	717.02	1.580
O–CH_2_	717.02	1.410
O–CH_3_	717.02	1.410
CH_2_–CH_3_	501.91	1.540

a The parameters are based on the description of DMMP and Agent.^32^

**Table 4:** Bond Bending Parameters for TMP and TEP^a^

Angle	*K*_a_, kcal/mol/rad^2^	θ_0_, deg
O=P–O	100.14	116.5
O–P–O	62.10	106.5
P–O–CH_2_	80.07	121.0
P–O–CH_3_	80.07	121.0
O–CH_2_–CH_3_	62.10	106.0

a The parameters are based on the description of DMMP and Agent.^32^

**Table 5:** Dihedral Parameters for TMP and TEP^a^

Angle	*K*_1_, kcal/mol	*d*_1_, deg	*K*_2_, kcal/mol	*d*_2_, deg	*K*_3_, kcal/mol	*d*_3_, deg	*K*_4_, kcal/mol	*d*_4_, deg
O=P—O—CH_2_	3.0502	90.0	–2.1901	–8.5943	0.5800	162.8114	0.7901	–25.7829
O=P—O—CH_3_	3.0502	90.0	–2.1901	–8.5943	0.5800	162.8114	0.7901	–25.7829
P–O–CH_2_–CH_3_	0.33941	90.0	0.54300	–2.5891	0.12091	174.82163	–0.361103	–7.76754

a The first two are based on description of DMMP and Agent.^32^ The last one is calculated using *ab initio* potential energy scan of ethyl phosphinate.

To recap, the LJ parameters were inherited from ref 32, except those of CH_2_, which were obtained from ref 35. The partial charges were computed by using the *ab initio* CHELPG analysis. The bond bending and stretching, and the dihedral parameters were obtained from ref 32, except the P–O–CH_2_–CH_3_ dihedral, which was fitted using an *ab initio* potential energy scan.

### Liquid Density

To assess the force field parametrization, first, the liquid densities of pure TMP and TEP were calculated using MD simulations. The simulations were performed in an isothermal–isobaric (*NPT*) ensemble with temperatures ranging from the melting point to the boiling point of the molecules at 1 bar pressure. These consisted of 150 molecules placed in a cubic box such that the size of the box was greater than twice the cutoff radius. This size was chosen to avoid nonphysical interactions due to periodic boundary conditions. The Nosé–Hoover thermostat and barostat were used with 100 and 1000 fs damping times, respectively. The system was simulated for 3 ns, of which 0.25 ns were used for equilibration, with each time step being 1 fs. The time integration was performed using the Verlet algorithm. The Lorentz–Berthelot mixing rules were used for nonbonded interaction parameters of unlike sites. A cutoff of 14 Å was used for nonbonded interactions, where the long-range electrostatic interactions were calculated using the Ewald summation with a tolerance of 10^–6^.^36^ Tail corrections were used for LJ interactions. All MD simulations were performed using the LAMMPS/20250204 ^37^ software.

### Vapor–Liquid Equilibrium

Gibbs ensemble Monte Carlo (GEMC)^38^ simulations were performed to determine the vapor–liquid equilibrium. In these simulations, the molecules are placed in two separate simulation boxes that are linked to each other. In addition to standard MC translation, rotation, and reinsertion moves within the same box, molecules are allowed to move from one box to another, and the volume in each box is adjusted such that the total volume in the two boxes remains constant. This allows one to simulate the vapor and liquid phases simultaneously. The pressure in the vapor phase was computed as the molecular pressure using the virial theorem. The initial densities and number of molecules in each box were chosen to ensure an appropriate sampling of the equilibrium properties of each phase. For the first box, there were 130 molecules with a cell length of 33 Å and 29 Å for TEP and TMP, respectively. Similarly, for the second box, there were 10 molecules with a cell length of 76 Å and 69 Å for TEP and TMP, respectively. The move probabilities were configured with 10% for Gibbs volume exchange, and the remaining move attempts were allocated with equal probability for translations, rotations, molecular reinsertions, and Gibbs particle swaps. The system was equilibrated for 10^5^ cycles, and production runs were performed for 2 × 10^5^ cycles. The cutoff for nonbonded interactions and the Ewald summation tolerance were chosen to match those used in the MD simulations. In addition, tail corrections were used for LJ interactions. RASPA 2.6.32 ^39^ was used to perform the GEMC simulations.

### Critical Parameters

The binodal curve is generated using the equilibrium density values calculated from GEMC simulations. These values can be used to calculate critical parameters, such as critical temperature and critical density.^32,33^ The Ising relation^40^ (eq 6) was used to extrapolate the densities in the vapor ρ_vap_ and liquid ρ_liq_ phases to calculate the critical temperature *T*_crit_. Similarly, the estimated critical temperature was used to calculate the critical density ρ_crit_ using the rule of rectilinear diameter (eq 7).^41^ Temperatures above 480 K were used for fitting the critical parameters.^32^ Here, *A* and *B* are fitting parameters.

6 $$\rho_{\mathrm{liq}}-\rho_{\mathrm{vap}}=B|T-T_{\mathrm{crit}}{|}^{0.325}$$

7 $$\frac{\rho_{\mathrm{liq}}+\rho_{\mathrm{vap}}}{2}=\rho_{\mathrm{crit}}+A\left(T_{\mathrm{crit}}-T\right)$$

### Enthalpy of Vaporization

The enthalpy of vaporization was estimated from MD *NPT* simulations by utilizing two separate simulation boxes to represent the vapor and liquid phases.^33^ The initial number of molecules *N*, densities, and pressures were set according to the values obtained from GEMC simulations. Vapor pressures below 400 K were extrapolated from a linear regression of GEMC data above 400 K, since the vapor pressures at those temperatures were scattered (Figure 6c). Additionally, at temperatures lower than 400 K, the Ewald summation tolerance was reduced to 10^–4^ and the Coulombic cutoff was increased to 20 Å for the vapor phase. Due to the large vapor box size (>100 Å) at lower temperatures, Ewald summation became a severe bottleneck in the simulations. By decreasing the tolerance and increasing the cutoff, there was a significant speed-up in the simulation time. It was ensured that these modifications did not have an effect on the fluctuations by looking at the percentage difference of the average potential energy and temperature, which were 0.03% and 0.18%, respectively, for the lowest temperature. The system was simulated for 5 ns, of which 1 ns was used for equilibration. The enthalpy of vaporization was calculated as:^33,42^

8 $$\Delta H_{\mathrm{vap}}=U_{\mathrm{vap}}-U_{\mathrm{liq}}+R_{g}T+\frac{1}{2}\left[R_{g}\left(\left\langle T_{\mathrm{liq}}\right\rangle -\left\langle T_{\mathrm{vap}}\right\rangle \right)(3N-6\right)]$$

where *U*_vap_ and *U*_liq_ are the average potential energies of the system, normalized by the number of molecules in the vapor phase and the liquid phase, respectively. The last term in eq 8 is a correction term, where ⟨*T*_vap_⟩ and ⟨*T*_liq_⟩ are the average temperature fluctuations in each phase. It is used since ⟨*T*_vap_⟩ can deviate from the temperature *T* set in the thermostat.^42^

### Surface Tension

The surface tension at the vapor–liquid interface was calculated in the canonical (*NVT*) ensemble using MD simulations, following the procedure outlined by Ivanova et al.^15^ Surface tension is defined for a flat vapor–liquid interface by the Irving–Kirkwood^43^ relation,

9 $$\gamma =\int_{-\infty }^{\infty }\left[P_{⊥}\left(z\right)-P_{∥}\left(z\right)\right]dz$$

The simulation consists of a symmetric slab of liquid surrounded by vapor, such that there are two vapor–liquid interfaces along the *z*-axis. This reduces the above equation to the following, where *P*_*zz*_ is the average pressure along the *z*-axis that represents *P*_∥_(*z*), the normal component. The tangential component, *P*_∥_(*z*), is represented by the mean of *P*_*xx*_ and *P*_*yy*_, the average pressure along the *x* and *y* axes. Since there are two interfaces, a factor of 1/2 is used

10 $$\gamma =\frac{1}{2}l_{z}\left[P_{zz}-\frac{1}{2}\left(P_{xx}+P_{yy}\right)\right]$$

11 $$\sigma \approx {\left(\frac{M}{\rho N_{A}}\right)}^{1/3}$$

To ensure that the slab has an adequate thickness, σ is used to estimate the molecular size of TEP and TMP. *M* is the molecular weight, ρ is the bulk liquid density, and *N*_A_ is Avogadro’s number. At standard conditions (Table 6), σ is estimated as 6.57 Å and 5.78 Å for TEP and TMP, respectively. For TMP, the simulation box was divided into two cubes, each with sides of 7σ, such that the box dimensions are 7σ × 7σ × 14σ. Similarly, a factor of 8σ was used for TEP. A larger factor was used for TEP to minimize errors due to its larger size. Figure 3 shows the resulting simulation box for TEP. The vapor and liquid densities obtained from GEMC simulations were used to calculate the number of molecules in each cube, and the molecules were placed randomly in the entire simulation box. During equilibration, the molecules form a slab of liquid with a well-defined vapor–liquid interface. This was followed by the calculation of the pressure tensor *P*_*xx*_, *P*_*yy*_, and *P*_*zz*_. The system was equilibrated for 4 ns, followed by production runs of 11 ns. The surface tension at absolute zero, γ_0_, was calculated using the following relation. Here, *n* is a fitting parameter.^31^

12 $$\gamma =\gamma_{0}{\left(1-\frac{T}{T_{\mathrm{crit}}}\right)}^{n}$$

**Table 6:** Density of Bulk Liquid (in kg/m^3^) of TMP and TEP Compared with Other organophosphates^33,54^ and Corresponding Literature Values at Standard Conditions (298.15 K and 1 atm) Calculated Using MD Simulations in the NPT Ensemble and Compared to the Experimental Values

	TEP	TMP	DMMP	DIMP	Agent
ρ_liq_ MD	1072 ± 1	1191 ± 4	1167 ± 4	985 ± 2	1104 ± 7
ρ_liq_ Exp	1064.2 ^20^	1205 ^19^	1156.0 ^51^	976.0 ^52^	1088.7 ^53^
Err. (%)	0.73	1.16	0.95	0.92	1.41

**Figure 3:**
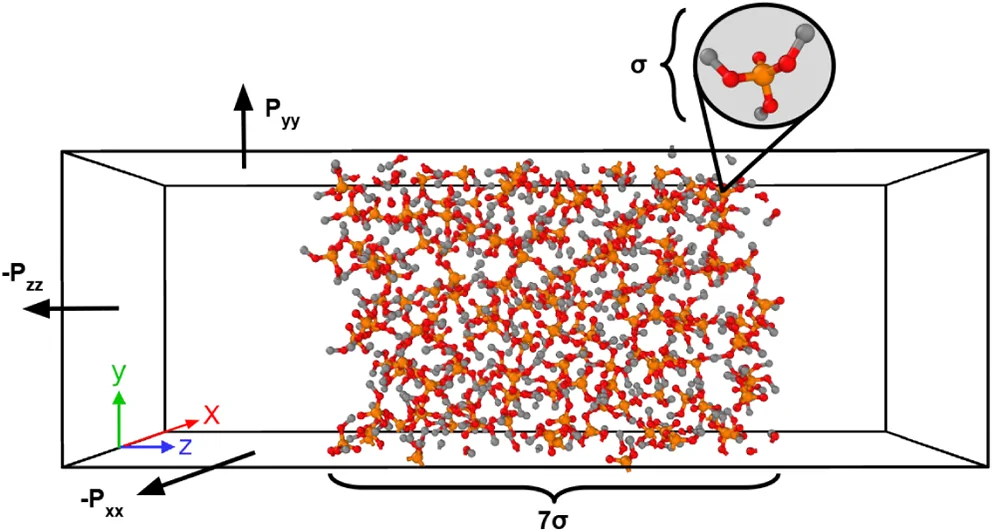
Snapshot of the vapor–liquid interface of TMP, along with computed pressures and molecular size σ.

### Viscosity

The shear viscosity of homogeneous fluids is commonly predicted using the Green–Kubo method (see eq 13).^44^ Following this approach, the shear viscosity depends on the volume of the fluid (*V*), the temperature in the ensemble (*T*), and the off-diagonal elements of the pressure tensor (*P*_*xy*_, *P*_*xz*_, and *P*_*yz*_), which are used to compute their autocorrelation (AC) and subsequent integration. The shear viscosity is calculated according to

13 $$\eta \left(t^{\prime }\right)=\frac{V}{3N_{\mathrm{rep}}k_{B}T}\overset{N_{\mathrm{rep}}}{\underset{n=1}{\sum }}\overset{3}{\underset{i}{\sum }}\int_{0}^{t^{\prime }}\langle P_{i,n}\left(0\right)P_{i,n}\left(t\right)\rangle_{t_{0}}dt,,i\in \left\{xy,xz,yz\right\}$$

where the factor 1/3 comes from averaging over the off-diagonal pressure components,nents, *N*_rep_ is the number of replicates (different initial configurations and velocities) of one state, ${\left\langle \cdot \right\rangle }_{t_{0}}$ is the average of the autocorrelated component over different time origins, and the integral is along the correlation time (not simulation time), with *t*′ → ∞.^44^ This approach has been used before to predict the viscosities of DMMP and DIMP under standard conditions.^17^

We calculated the shear viscosity of TMP and TEP following three subsequent steps: (1) the state of our system is defined from our density predictions at 1 bar and a chosen temperature (i.e., from 258 K to 337 K for TMP, as well as from 290 K to 342 K for TEP), (2) the system is equilibrated in an *NVT* ensemble for 40 ps, and (3) we perform simulations in the *NVE* ensemble to calculate the AC of each off-diagonal pressure component every *N*_f_ steps (*N*_f_ × δ*t* is the correlation time, where δ*t* is the simulation time step). Hence, every *N*_f_ steps, we have a new time origin. The steps from 1 to 3 are repeated *N*_rep_ times. (4) The resulting AC functions are averaged over the off-diagonal *P*_*i*_ elements, time origins, and *N*_rep_. Finally, (5) the averaged AC is integrated along increasing time lags until the correlation time.

As the integration in eq 13 goes to infinity, we must ensure that *N*_f_ is large enough to ensure convergence of our averaged AC to zero. After preliminary calculations (see Supporting Information), we chose *N*_f_ = 4 × 10^4^ for a time step δ*t* = 1 fs, with *N*_r_ = 4 × 10^3^ time lags (where the smallest time lag is Δ*t* = 10 fs) for each correlation time, and 500 time origins, as well as 10 replicates. Hence, we simulated each replicate for 20 ns. Also, since the Green–Kubo method predicts size-independent shear viscosities, we set *V* = (2*r*_cut_)^3^, where *r*_cut_ is the cutoff radius, and adjusted the number of molecules accordingly.

We curve-fitted the resulting viscosity from eq 13 to estimate the shear viscosity at an infinite correlation time, according to

14 $$\eta \left(t^{\prime }\right)=\eta_{\infty }\left\{1-\exp\left[-{\left(t^{\prime }/\tau \right)}^{\beta }\right]\right\}$$

where η_∞_, τ, and β are fitting parameters that relate to infinite-time viscosity, decay time, and the exponent of slow relaxation, respectively.

Finally, to estimate the viscosity as a function of temperature, we curve-fitted our resulting η_∞_ at each temperature according to the Andrade equation^45^

15 η(T)=A⁡exp(B/T)

where *A* and *B* are the fitting parameters related to the effective molecular size and the energy required to move to free spaces, respectively.^46,47^

### Self-Diffusivity

Unlike our procedure to predict the viscosities of TMP and TEP, the self-diffusion coefficient is quite sensitive to the simulation box size when using the Einstein approach,

16 $$D_{L}=\frac{1}{6}\underset{t\to \infty }{\lim}\frac{d}{dt}\left\langle \frac{1}{N}\overset{N}{\underset{\alpha =1}{\sum }}\right|{r_{\alpha }(t)-r_{\alpha }(0)|}^{2}\rangle$$

where *N* is the number of molecules in the simulation box, and ***r***_α_ is the position of the αth molecule’s center of mass. The angle brackets refer to the mean squared displacement (MSD). To avoid such box-size dependence, we used a method proposed by Yeh and Hummer^48^

17 $$D=D_{L}+\frac{1}{L}\frac{k_{B}T\zeta }{6\pi \eta }$$

where ζ ≡ 2.837297. We equilibrated our system in an *NVT* ensemble for 100 ps, with a Nosé-Hoover thermostat, in a box of size *L* = 2*r*_cut_, at a corresponding range of temperatures (from 258 K to 337 K for TMP, and from 290 K to 342 K for TEP), and derived the number of particles based on *L* and our density predictions at 1 bar. Then, we performed a production run of 5 ns in an *NVE* ensemble to calculate *D*_*L*_ from eq 16. This approach reduces computational time compared to other methods.^44,49^ Then, we linearly extrapolate our resulting *D*_*L*_ to an infinite box size by using a correction term (second term, right-hand side of eq 17). To perform the extrapolation, we plot *D* = *D*(1/*L*). The slope is *k*_B_ *T*ζ/6πη, and *D*_*L*_ = *D*(1/2*r*_cut_). Therefore, the size-independent self-diffusion coefficient, *D*_∞_, is predicted at 1/*L →* 0 (infinitely large box size). This approach requires prior knowledge of the fluid’s viscosity.

As with our approach for predicting viscosities, we must simulate long enough to reach the diffusive (linear) regime. Also, although the resulting *D*_*L*_ calculations are less noisy than the viscosity predictions, it is still worth running multiple replicates and averaging the resulting *D*_*L*_. For TMP and TEP, we used 60 replicates. Our procedure for finding the diffusive regime is described in the Supporting Information. We calculated the MSD values and *D*_*L*_ using the PyLAT toolkit.^50^

## Results and Discussion

The TraPPE force fields for TMP and TEP were parametrized by transferring LJ, bond, angle, and dihedral terms from other organophosphates,^33^ and the partial charges were obtained using the CHELPG analysis. The resulting partial charges are shown in Tables 1 and 2. The P–O–CH_2_–CH_3_ dihedral in TEP was parametrized using an *ab initio* relaxed potential energy scan, which was performed on ethyl phosphinate (Figure 1e). At each step of the scan, the molecule was relaxed, excluding the fixed dihedral. To account for this, we subtracted the LJ, electrostatic, bond, angle, and other remaining dihedral energy contributions at each step. Finally, the result relative to the minimal energy conformation was used to fit eq 5 (shown in Figure 4) to obtain the dihedral parameters for P–O–CH_2_–CH_3_ (shown in Table 5).

**Figure 4:**
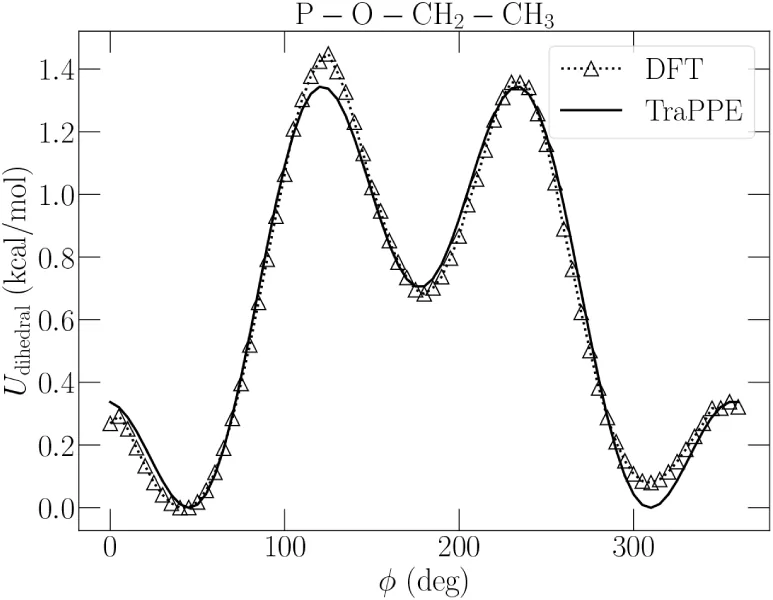
Energy contribution of the dihedral angle P–O–CH_2_–CH_3_ calculated based on *ab initio*, and fit to the TraPPE dihedral potential (eq 5).

### Liquid Density

The performance of the force field models was first validated by calculating bulk liquid densities in the MD *NPT* ensemble. Table 6 shows the liquid densities of bulk TMP and TEP compared with literature data under standard conditions. It was also compared with results from TraPPE force fields for DMMP, DIMP, and Agent along with their corresponding literature values. The resulting density of TEP was close to the literature value, while TMP underestimated the experimental density by 1.2%. This was consistent with other TraPPE organophosphates, which have 1% errors.

Figure 5 shows the liquid density of TMP and TEP between 280 K and 350 K at 1 bar. The results are compared with experimental data.^19,20^ For TEP, the liquid densities based on MD simulations using OPLS all-atom (OPLS/AA) and GAFF force fields are shown^28^ along with literature data obtained from handbooks.^31,55,56^ For TMP, density was also compared with literature that utilizes vendor-supplied values.^57^ Liquid densities of other similar organophosphates, such as DIMP, DMMP, and Agent based on TraPPE, are also shown.^32^ The DIMP densities presented here have been recalculated using the corrected input parameters established in ref 54, replacing the incorrect values from ref 32. The liquid densities of TMP and TEP are reported in a wider range of temperatures in the Supporting Information.

**Figure 5:**
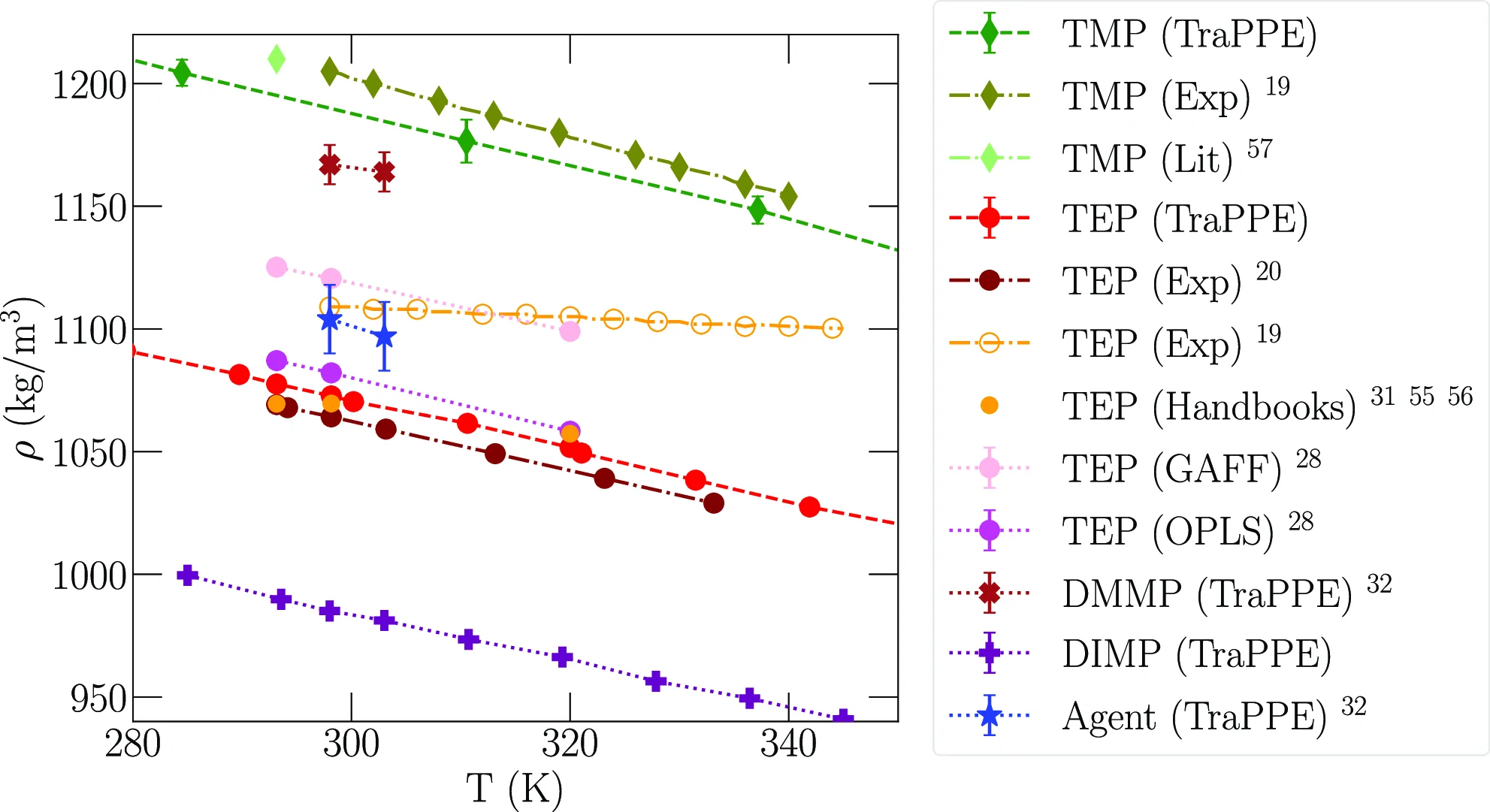
MD predictions for the density of bulk liquid TMP and TEP using the TraPPE force field. Compared with experimental data for TMP and TEP,^19,20^ literature data based on handbooks for TEP,^31,55,56^ literature data for TMP,^57^ liquid densities based on OPLS/AA and GAFF force fields for TEP,^28^ and liquid densities of similar organophosphates based on the TraPPE force field.^32^ Note that the DIMP densities were recalculated based on corrected force fields reported in ref 54. Here, the error bars represent twice the square root of the variance, which is estimated by blocking analysis.

The calculated liquid densities of both TMP and TEP agree well with experimental data^19,20^ with 1–2% error, which is in line with the MD predictions for DMMP and DIMP using the TraPPE force field.^32^ However, there is a noticeable difference between the density of TEP calculated using MD and the experimental density from ref 19. We observe that the experimental data from ref 19 for TEP does not agree with literature OPLS/AA and GAFF force field models and also the reported handbook values. This disagreement was also pointed out in the experimental study by ref 20 who chose to reject it in their comparison. Moreover, the slope of the density–temperature dependence from ref 19 does not agree with the slope of any of the other organophosphates shown in Figure 5. It suggests that the TEP liquid densities reported in ref 19 have a systematic error, and one should rather rely on the density reported in ref 20. The densities calculated from our MD simulations agree well with ref 20.

### Vapor–Liquid Equilibrium

Figure 6 shows the vapor–liquid equilibrium of TMP and TEP obtained from GEMC simulations. Figure 6a shows the binodals, compared to the Agent calculated using GEMC.^32^ Here, eqs 6 and 7 were used to fit the critical points and generate smooth VLE trends. The critical parameters obtained are shown in Table 7. We observe that the critical point of TEP is the highest among all the molecules shown here. The liquid densities of TEP at lower temperatures have better agreement with the Agent compared to other organophosphates. Figure 6b provides a closer look at the vapor phase section of the VLE. The values of densities below 350 K are not shown, as at these temperatures the vapor phase densities are effectively zero (<10^–1^ kg/m^3^) and can be ignored.^32,58^ Here, the vapor density of TMP appears very close to the vapor pressures of the Agent across the entire range of temperatures. The large error bars for TEP predictions around 400 K can be attributed to insufficient sampling due to its larger molecular size and lower vapor density compared to TMP.

**Figure 6:**
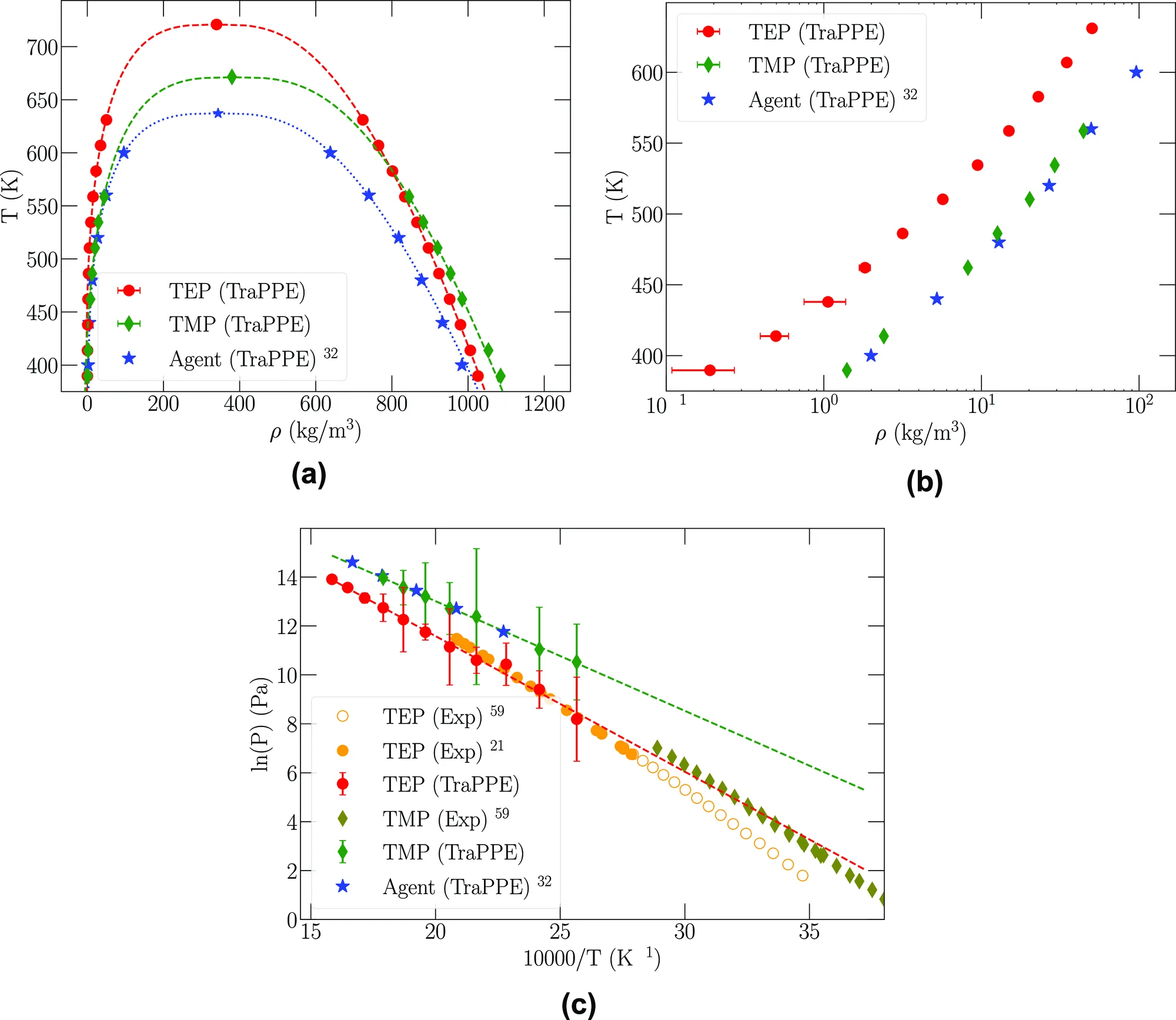
Vapor–liquid equilibrium of TMP and TEP predicted using GEMC simulations. (a) Vapor–liquid binodal, (b) the semilog plot of the vapor phase, and (c) the Clausius–Clapeyron relation along with its linear fit are compared with experimental data.^21,59^ The data for TEP and TMP is shown along with the MC predictions for Agent.^32^ The error bars in Figure 6a and 6b represent twice the standard deviation. The error bars in Figure 6c represent the standard error of the mean.

**Table 7:** Critical Temperature, *T*_crit_, and Critical Density, ρ_crit_ of TMP and TEP Obtained from GEMC Simulations^a^

Molecule	*T*_crit_ (K)	ρ_crit_ (kg/m^3^)	γ_0_ (mN/m)
TMP	671.2	383.6	63.1
TEP	720.9	339.3	63.8

a The surface tension at 0 K, γ_0_, predicted from extrapolating the MD simulation data for γ­(*T*).

Figure 6c shows the vapor pressures in the Clausius–Clapeyron relation for TMP and TEP with their linear regression, and it is compared with experimental data.^21,59^ TEP shows good agreement with experimental values at higher temperatures and is within the error bars. As the experimental data for TMP was not available at higher temperatures, we cannot make a direct comparison between GEMC predictions and experimental data. However, we can observe that the slope between the extrapolated GEMC predictions and experimental data is different.

### Enthalpy of Vaporization

Figure 7 shows the enthalpy of vaporization of TMP and TEP predicted in the MD *NPT* ensemble using eq 8 at temperatures ranging from 270 K to 610 K compared to experimental data.^21,60^ For TEP, the liquid densities based on MD simulations using OPLS/AA and GAFF force fields are also shown.^28^ The vapor and the liquid phases were simulated separately, with the initial density and pressure set based on results from GEMC simulations. For temperatures lower than 400 K, initial pressures were obtained from linear regression. To speed up the simulations in the vapor phase, the parameter for Ewald summation was reduced and the Coulombic cutoff was increased. There is only a single experimental data point for TMP, which aligns well with the predicted values.^60^ Our predictions for the enthalpy of vaporization of TEP exceed the data point from ref 60. They agree well with the data from ref 21 at lower temperatures, deviating at temperatures above 400 K. Enthalpy of vaporization usually decreases monotonically with an increase in temperature, which is consistent with the predicted values, unlike the experimental data from ref 21 at high temperatures. The predictions from OPLS/AA and GAFF force fields^28^ for TEP are much higher compared to our predictions and other literature predictions.^21,60^ Additionally, we estimated the enthalpy of vaporization of TEP and TMP from GEMC simulations at 500 K using a linear regression based on the Clausius–Clapeyron equation.^32^ Only temperatures ranging from 460 K to 535 K were used for the estimation. These data points are shown with crosses in Figure 7 and slightly overpredict the results based on MD.

**Figure 7:**
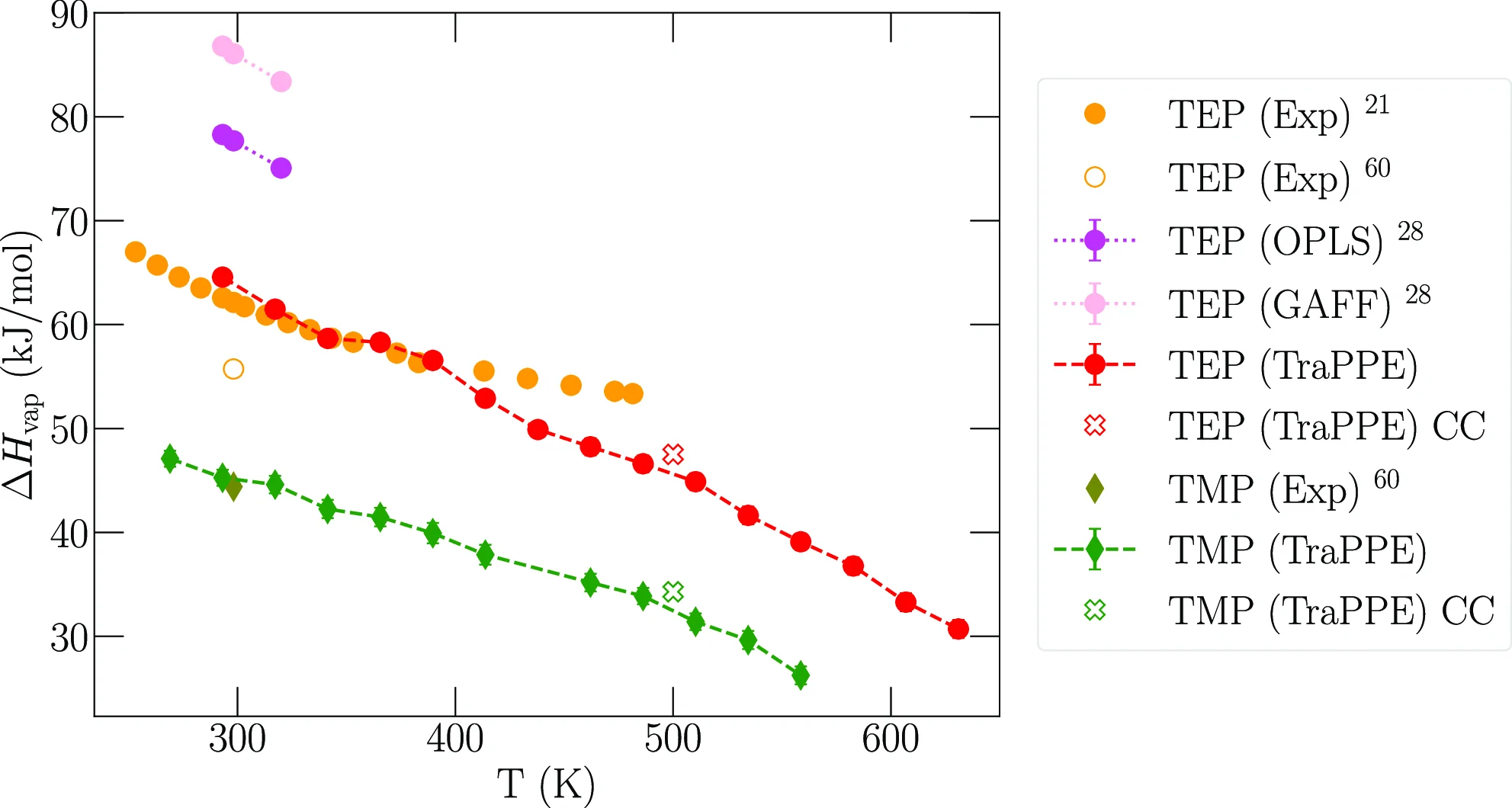
MD predictions of the enthalpy of vaporization of TMP and TEP with experimental measurements,^21,60^ and MD predictions based on OPLS/AA and GAFF force fields for TEP.^28^ The error bars represent twice the square root of the variance, which is estimated by blocking analysis. The estimate from the linear regression based on the Clausius–Clapeyron (CC) relation is also shown.

### Surface Tension

Figure 8 shows the surface tension of TMP and TEP at temperatures ranging between 270 K and 400 K, predicted by simulating the vapor–liquid interfaces in a symmetric slab of fluid molecules in the canonical ensemble. Equation 11 was used to estimate the molecular size, which was used to estimate the system volume. This was done to ensure that the system forms a liquid slab with sufficient thickness. After equilibration, the tangential and normal components of pressure tensors were averaged and the surface tension was calculated using eq 10. Figure 8a shows the comparison with experimental data,^22,23^ along with literature data from handbooks.^31^ It is also compared with MD predictions of other organophosphates, such as DMMP, DIMP, and Agent^15^ (Figure 8b), and MD predictions for TEP using OPLS/AA and GAFF force fields.^28^ The DIMP surface tensions presented here have been recalculated using the corrected input parameters established in ref 54 replacing the incorrect values from ref 15. The predicted values for TMP and TEP both consistently underestimate the experimental and literature data by ≈5 mN/m. However, we observe that the predicted values for TEP are consistent with DIMP and Agent. The surface tension predicted at 0 K calculated using eq 12 is shown in Table 7. This can be used to extrapolate surface tension at different temperature ranges.

**Figure 8:**
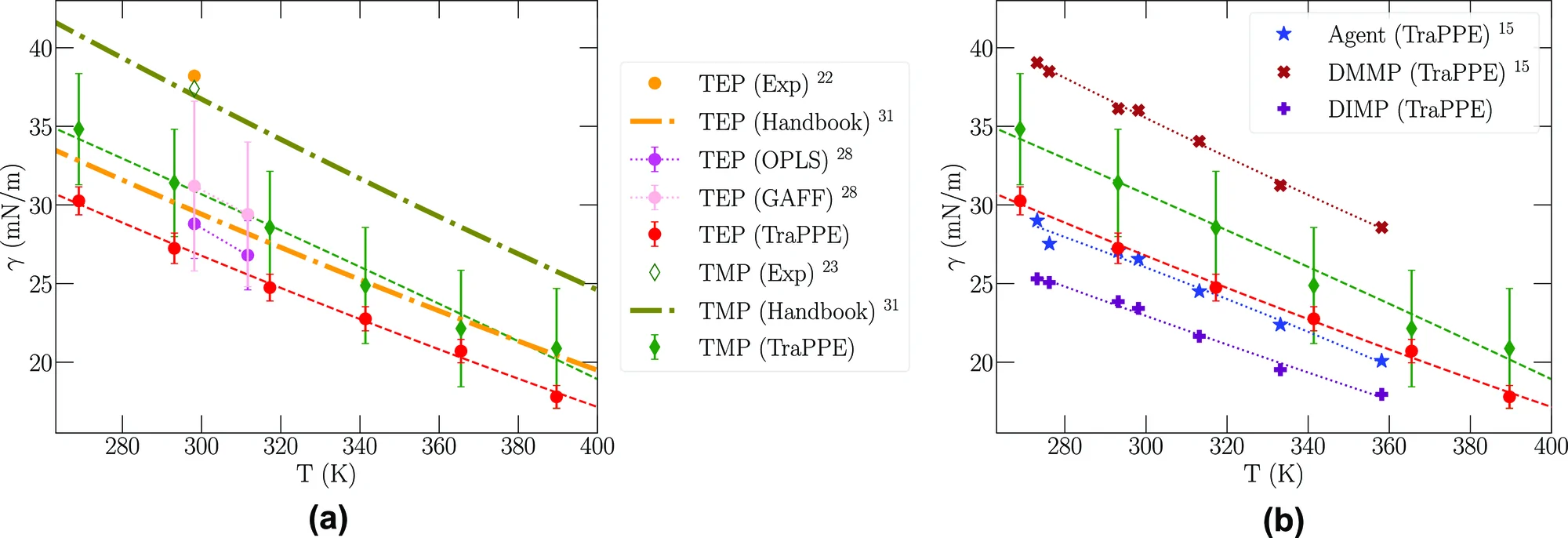
(a) MD predictions of the surface tension of TMP and TEP compared with experimental^22,23^ and literature data from a handbook,^31^ which provide parameters for eq 12. Comparison with OPLS/AA and GAFF predictions are also also shown.^28^ (b) Comparison of MD predictions for TMP and TEP with Agent, DMMP, and DIMP.^15^ Here, the surface tension of DIMP was recalculated based on corrections to the force field in ref 54. The error bars represent twice the square root of the variance, which is estimated by blocking analysis.

### Viscosity

Viscosities of TMP and TEP were predicted using the Green–Kubo approach in an *NVE* ensemble. The number of molecules was calculated from our density predictions at 1 bar, with temperatures ranging from 258 K to 337 K for TMP and from 290 K to 342 K for TEP. The resulting viscosities were curve-fitted following eq 14 and the Andrade equation (eq 15). Figure 9 shows the predicted viscosities of TMP and TEP, along with values collected from the literature.^19,24,25^ For a better comparison, predictions of the Agent from MD simulations are also included.^17^

**Figure 9:**
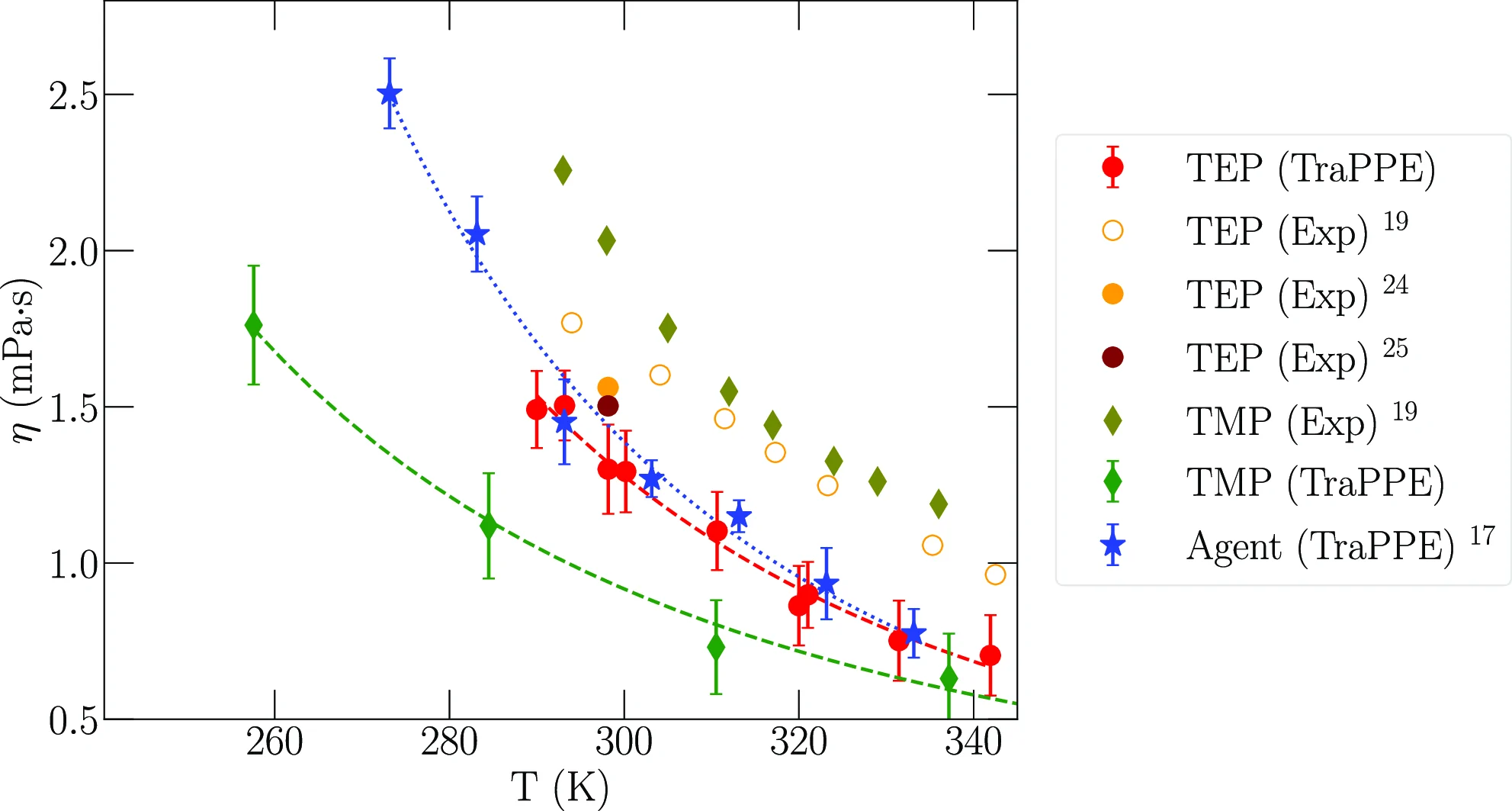
Viscosities of TMP and TEP at 1 bar, along with their values from the literature.^19,24,25^ MD predictions of the Agent are also shown for comparison.^17^ The error bars were estimated using the bootstrap method, accounting for 95% of confidence.

According to our prediction for TEP at 300 K, there is good agreement with experimental results,^24,25^ as well as with the viscosity of the Agent.^17^ This implies that TEP can play a comparable role as a simulant, even better than TMP, when focusing on viscosity studies. Compared with Kannan and Kishore’s results, our predictions for both TMP and TEP underestimate their measurements.^19^

According to the free volume theory, the molecular size plays an important role in the viscosity behavior of fluids. The higher the effective molecular diameter (σ), the higher the viscosity of the fluid. This is mainly due to a reduced freedom of the molecule to perform rotations and translations, as well as stronger intermolecular interactions.^46,47,61^ TMP (formed by P–O–CH_3_ branches) is smaller in size than TEP (formed by P–O–CH_2_–CH_3_ branches), so TMP should have lower viscosity values than TEP (as well as the Agent if we used the same approach). Our results are consistent with this observation. Similarly, the decrease of η = η­(*T*) should be steeper as the molecule is larger. This is a behavior that can be clearly seen in the viscosities of linear alkanes when compared with different chain lengths.^61^ However, the experimental measurements from Kannan and Kishore do not show this behavior between TMP and TEP; their results for TMP show a steeper decrease of η compared to TEP. A possible reason for this discrepancy could be the purity of their samples. It is essential to note that the presence of small impurities in samples can strongly affect the resulting viscosity measurements, even when samples have a purity of ∼95%.^18^

### Self-Diffusivity

The viscosities of TMP and TEP predicted in this work allowed us to use the approach from Yeh and Hummer (eq 17) to estimate the size-independent self-diffusivity (*D*_*∞*_) of TMP and TEP.^48^ Hence, it is sufficient to calculate *D*_*∞*_ using only one simulation box size. Figure 10 shows the predicted self-diffusivities of TMP and TEP after following this approach.

**Figure 10:**
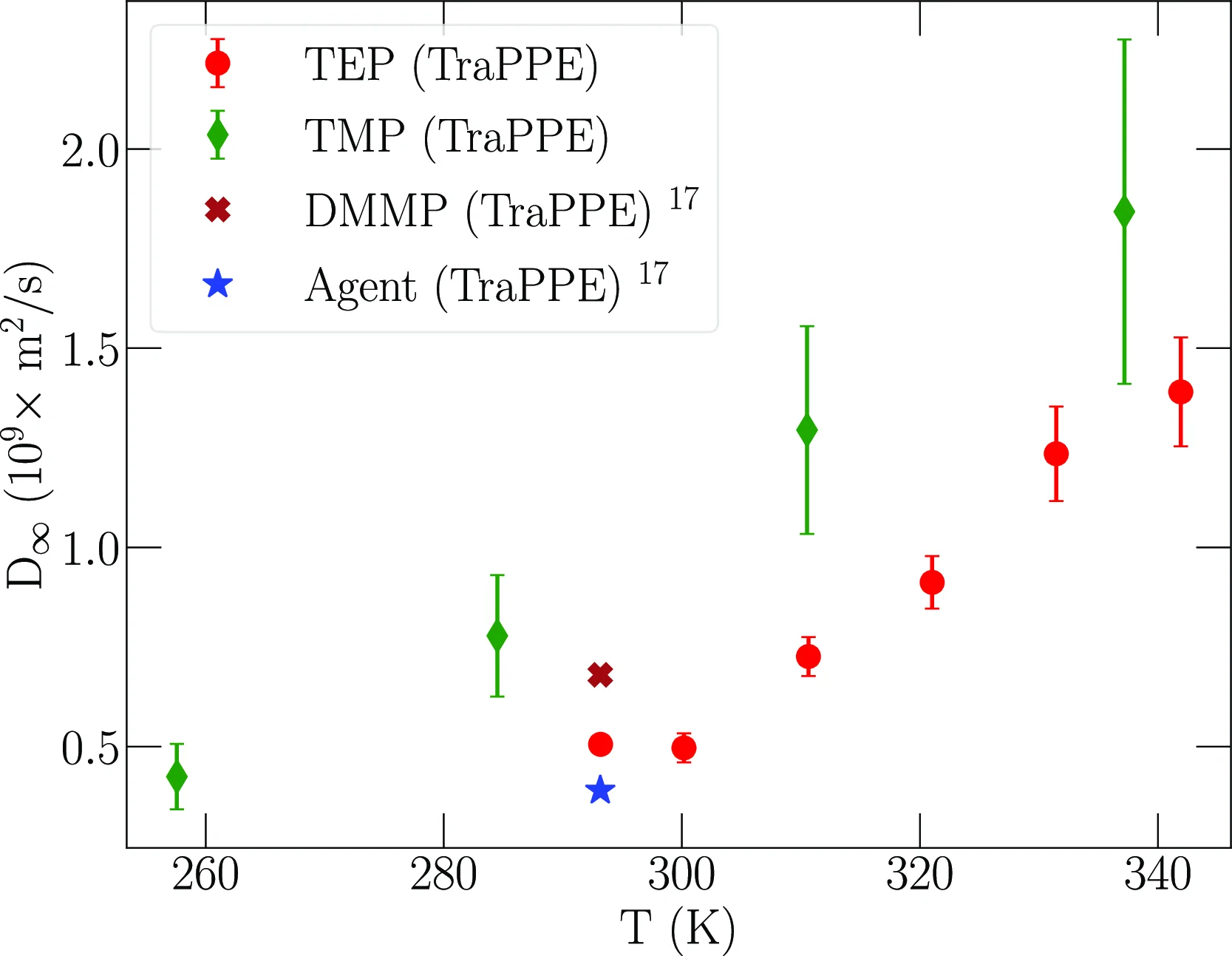
Self-diffusivities of TMP and TEP at 1 bar and different temperatures, along with previous MD predictions of DMMP and the Agent.^17^ The error bars represent twice the square root of the variance, which is estimated by blocking analysis.

To date, experimental data for the self-diffusivity of TMP and TEP has yet to be reported in the literature. Therefore, our analysis is only focused on comparing them with other organophosphates, such as DMMP and the Agent. Similar to the viscosity of TEP, the self-diffusion coefficient of TEP approaches the Agent’s coefficient. Moreover, TEP’s self-diffusivity yields results closer to the self-diffusivity of the Agent than DMMP. Therefore, for studies in self-diffusivity, TEP can be quite a promising simulant.

Focusing on the self-diffusivity of TMP, we show that it is the highest. The self-diffusivity of a fluid is intrinsically related to its viscosity. If a fluid shows high diffusivity (as is the case with TMP), it will very likely show a low viscosity. Our predictions on self-diffusivity are consistent with our predictions on viscosity.

## Conclusion

In this work, we considered triethyl phosphate, TEP, as an alternative CWA simulant. We extended the TraPPE force field to predict the thermodynamic and transport properties of TEP using Monte Carlo and molecular dynamics simulations. Comparison of the properties of TEP with data of other CWA simulants and the Agent itself helped assess its potential as a simulant.

Our predictions for the liquid density of TEP were in good agreement with experimental and literature data.^20,28,31,55,56^ Additionally, unlike the earlier MD predictions using the OPLS and GAFF force fields,^28,30^ we were able to reproduce the experimental enthalpy of vaporization and vapor pressures,^21^ and predict viscosities reasonably close to the available experimental data.^24,25^ Our predictions of surface tension were noticeably lower than the only experimental point in the literature^22^ and relatively close to the handbook data.^31^ Based on the previous success of the TraPPE force field in predicting surface tensions of organophosphorus compounds,^15^ this likely suggests that more systematic experimental measurements are needed.

Overall, our predictions and comparative analysis not only complemented the scarce literature data for TEP but also helped to identify inconsistencies in experimental measurements. The experimental liquid densities of TEP from ref 19 overestimate those from other literature sources^20,28,31,55,56^ and our predictions. Similar inconsistencies from ref 19 were found in their viscosity measurements, where the viscosities of TEP were reported to be lower than those for TMP. The opposite trend is expected since TEP has a larger molecular size than TMP, which should result in lower viscosities according to the free volume theory, and this is consistent with our predictions. Our viscosity predictions matched well with the most recent measurement of viscosity reported by Warmińska and Ṡmiechowski.^25^ Overall, the liquid density, viscosity, and surface tension of TEP are very close to those of the Agent, making TEP a promising simulant for applications related to aerosols.

Additionally, the transferability of the TraPPE force field allowed us to extend our study to another organophosphorus compound with a similar structure, and trimethyl phosphate, TMP. Predictions of liquid densities and enthalpies of vaporization of TMP are in good agreement with experimental data.^19,60^ Other data for TMP are very scarce, and the reported values of surface tension and viscosity can be considered as predictions, which however deviate from the properties of the Agent. At the same time, the vapor pressure and enthalpy of vaporization of TEP are very close to those of the Agent.

Overall, our work helps advance data for research on organophosphate CWAs, providing an assessment of emerging simulants for agents. TMP is a good simulant to model the vapor phase, while TEP is suitable for the liquid phase. Our work extends the known literature data across a wide range of temperatures and pressures. The force field developed here can be further used for other applications of molecular simulations, such as the adsorption of CWA simulants.^5,27,62^

## Supplementary Material





## Notes

Details about the viscosity and self-diffusivity calculations, and an extended range of calculated densities of the two organophosphates (PDF)
Input files (RASPA and LAMMPS formats) and scripts; data files for Figures 4, 5, 6, 7, 8, 9, 10 (ZIP)
