# Supplementary material for: Molecular Modeling of Triethyl Phosphate: Extension and Validation of the TraPPE Force Field
Source: J Phys Chem B. 2026 Aug 11;130(34):8686–97. doi: 10.1021/acs.jpcb.6c03259 (PMC13528294; doi:10.1021/acs.jpcb.6c03259)
Supplement: Supplementary file 1 [file jp6c03259_si_001.pdf]

Supporting Information for "Molecular Modeling of Triethyl Phosphate: Extension and Validation of the TraPPE Force Field"

Geordy Jomon,† Santiago A. Flores Roman,† Andrei L. Kolesnikov,‡,‡ and Gennady Y. Gor*,†

†*Otto H. York Department of Chemical and Materials Engineering,*
New Jersey Institute of Technology,
Newark, New Jersey 07102, United States
‡*Institut für Nichtklassische Chemie e.V.*
Leipzig 04318, Germany

E-mail: gor@njit.edu

Liquid Densities

Figure S1 shows the liquid densities predicted using MD NPT simulations for TMP and TEP. The densities are predicted at 1 atm pressure, from 200−500 K for TEP and 100−600 K for TMP. The simulation configuration is outlined in the main text.

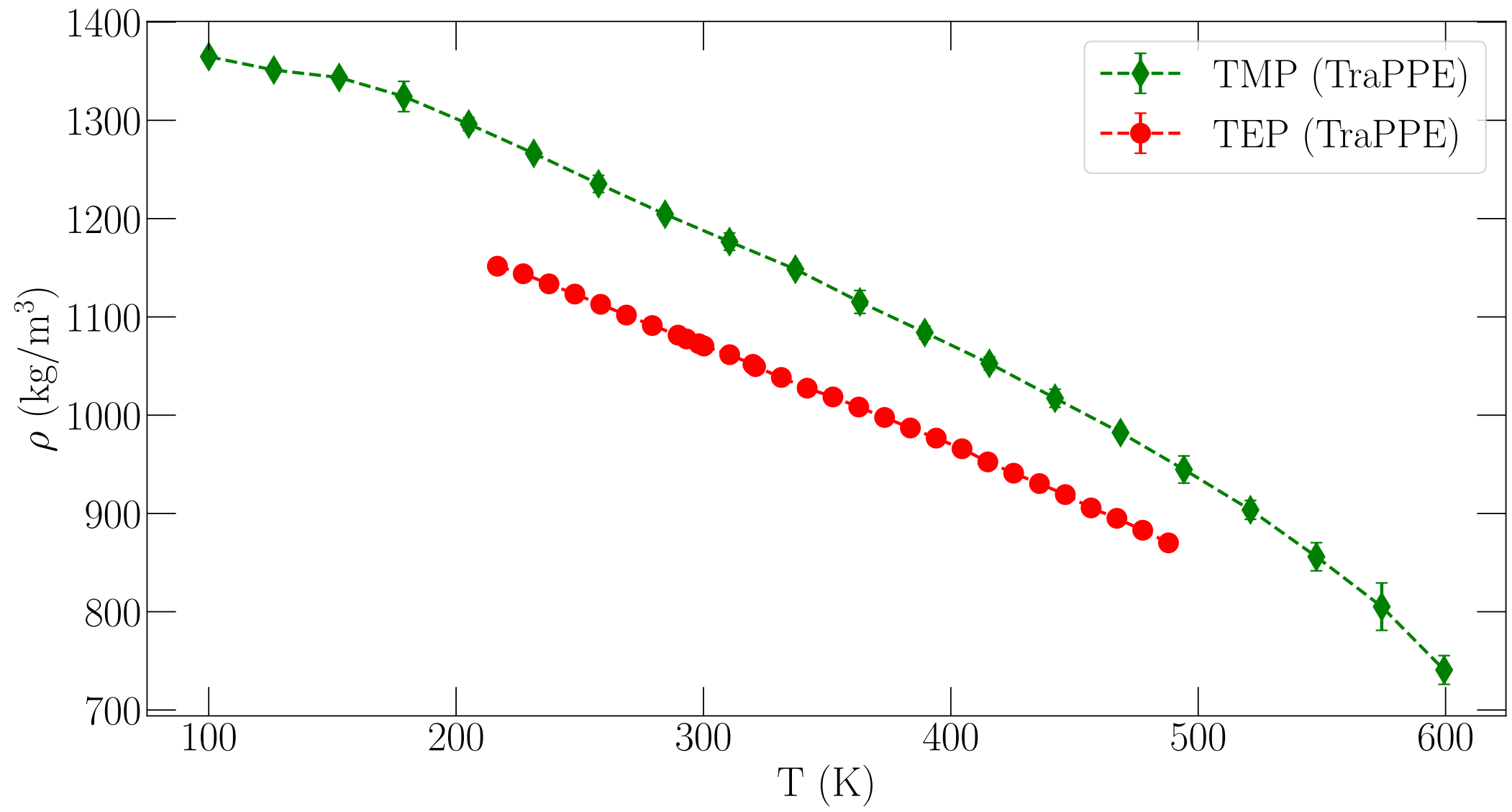

FIGURE S1: Liquid densities for TMP and TEP predicted using MD NPT simulations at wider range of temperatures.

Choice of Correlation Length to Predict Viscosity

Figure S2 shows the autocorrelation (after averaging over the off-diagonal pressures, multiple time origins and replicates) of TMP at 258 K and 337 K, along with its integration over increasing time-lags (Δt). Similarly, Figure S3 shows the averaged and integrated pressure autocorrelation (PAC) of TEP at 290 K and 342 K. The temperatures chosen are the lowest and highest used to calculate the viscosities of TMP and TEP. To correctly predict the viscosity of any fluid using the Green-Kubo approach, it is important to find a correlation length $N_{\mathrm{f}} \times \delta t$ long enough to ensure convergence of the PAC to zero. As a consequence, the viscosity (after integrating the PAC) must reach a plateau. The behavior of the PAC depends on the external temperature —the higher the temperature, the faster the convergence. Hence, an analysis most be made for each temperature. For low temperatures (below 280 K for TMP and 320 K for TEP), the convergence of the PAC is reached around 20 ps, and the plateau is ensured if the correlation time is set at least to 40 ps; for high temperatures, convergence is reached aroung 10 ps and the plateau is ensured if the correlation time is set at least to

20 ps.

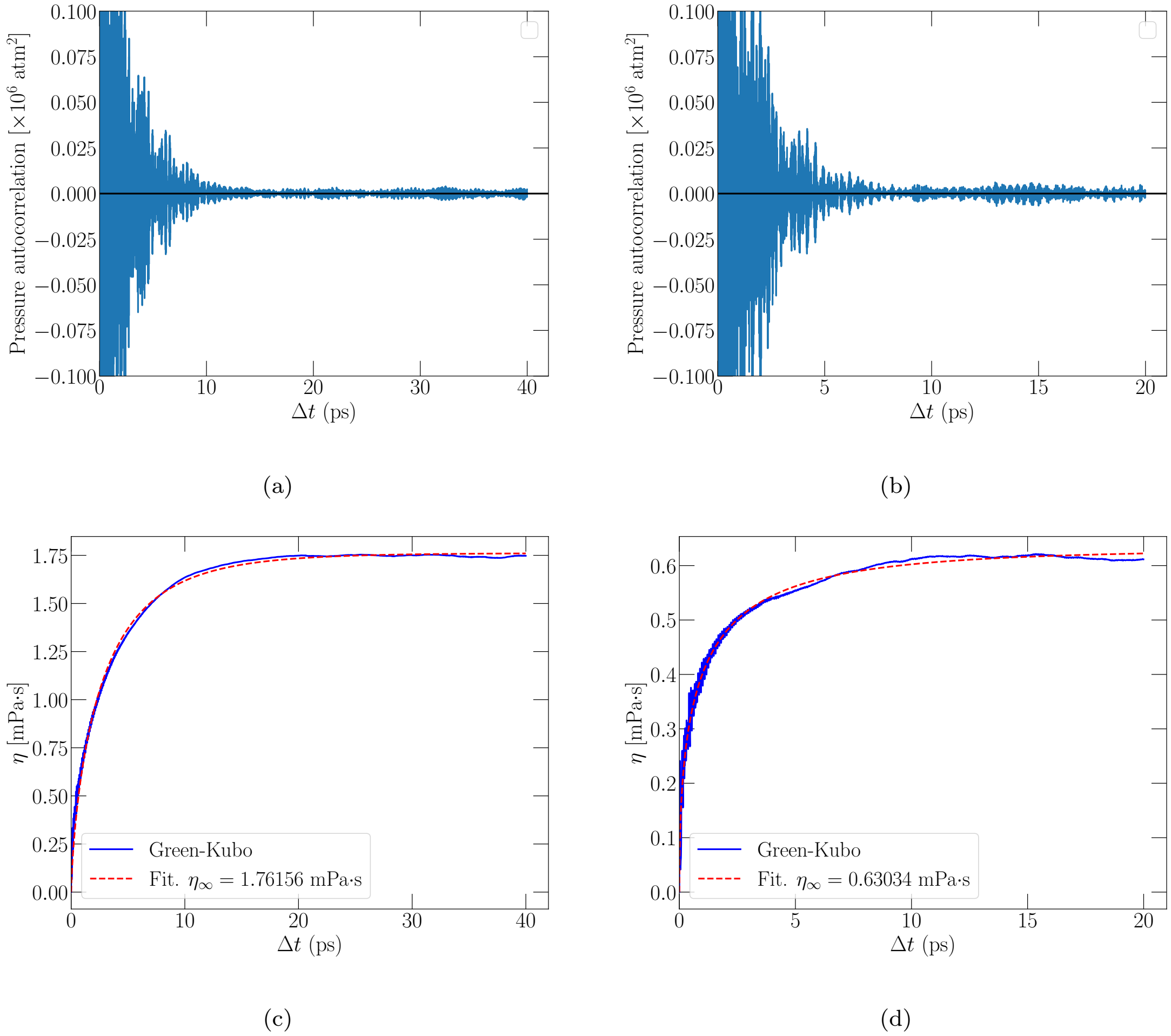

FIGURE S2: Averaged pressure autocorrelations (top) and their integrations over increasing time-lags (bottom) of TMP at 258 K (left) and 337 K (right). The red dashed lines show the curve fit to the resulting integration. η_∞ is the infinite-time viscosity.

Diffusive Regime for Self-Diffusivity Calculations

According to the Einstein approach, a correct calculation of D_L is made only in the diffusive regime, which can be found by calculating the MSD as a function of simulation time.

Figure S4 shows the MSD as a function of time in a log-log scale for TMP and TEP. According to Maginn et al., the diffusive regime can be identified as a linear region with a slope $\sim 1\,\text{Å}^2/\text{ps}$ in a log-log scale.[1] Therefore, simulations must be long enough to fall into this region but not so long as to capture noise. The identified region was from 100 ps − 1000 ps

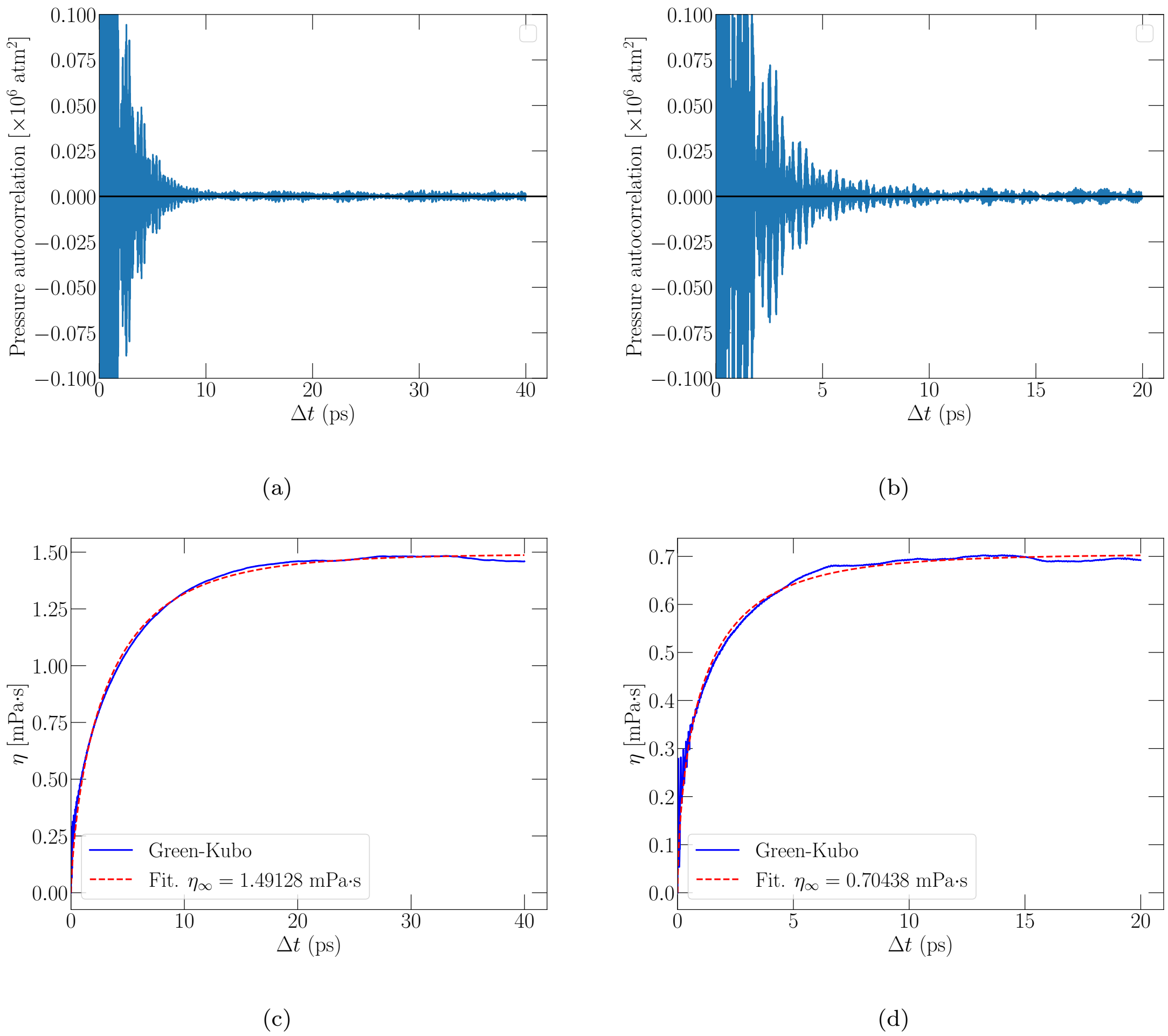

FIGURE S3: Averaged pressure autocorrelations (top) and their integrations over increasing time-lags (bottom) of TEP at 290 K (left) and 342 K (right). The red dashed lines show the curve fit to the resulting integration. η_∞ is the infinite-time viscosity.

for TMP, and from 200 ps − 2000 ps for TEP. In addition to this analysis, PyLAT searches for this region before calculating D_L.[2]

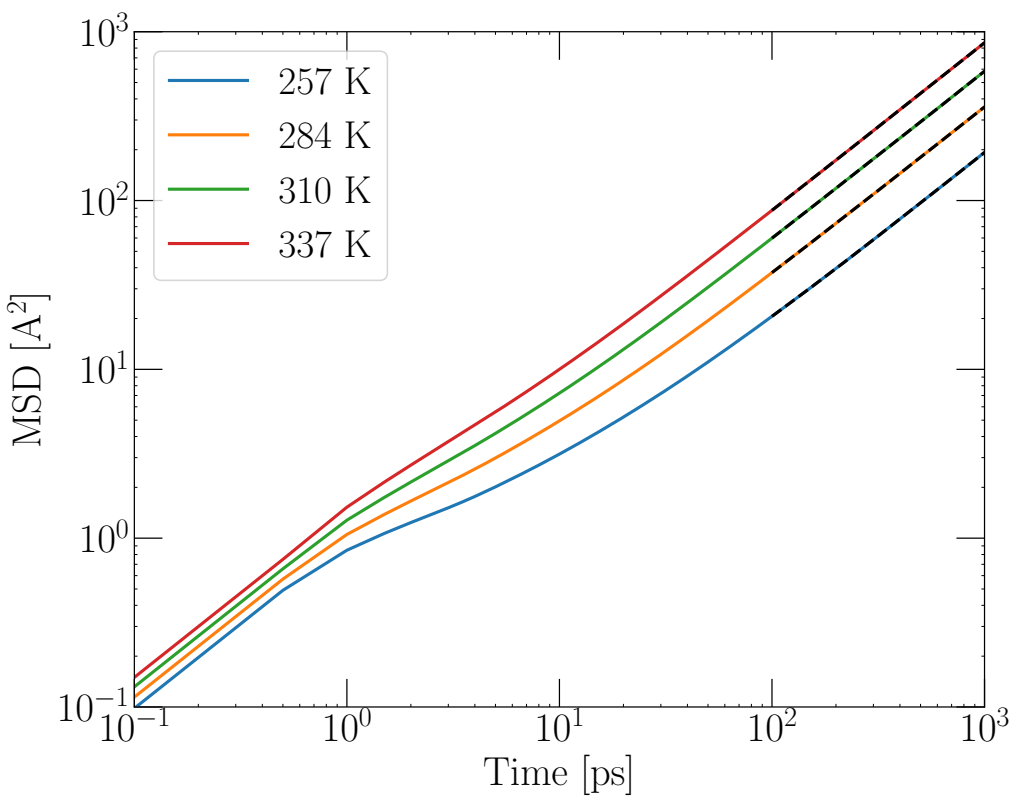

(a)

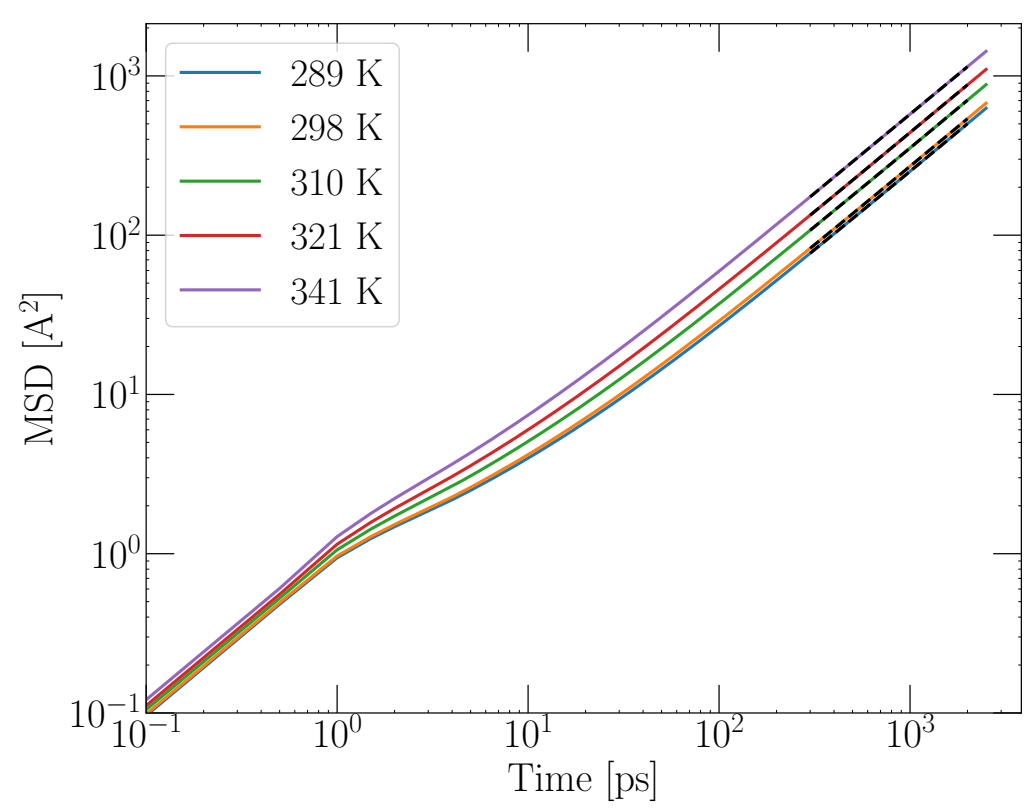

(b)

Figure S4: Mean square displacements (MSD) of (a) TMP and (b) TEP as a function of time for different temperatures. Dashed lines show the diffusive regime.

References

(1) Maginn, E. J.; Messerly, R. A.; Carlson, D. J.; Roe, D. R.; Elliot, J. R. Best Practices for Computing Transport Properties 1. Self-Diffusivity and Viscosity from Equilibrium Molecular Dynamics [Article v1.0]. *Living J. Comp. Mol. Sci.* **2019**, *1*, 6324–6324.

(2) Humbert, M. T.; Zhang, Y.; Maginn, E. J. PyLAT: Python LAMMPS Analysis Tools. *J. Chem. Inf. Model.* **2019**, *59*, 1301–1305.
